# Clinical and electrophysiological characteristics of Efavirenz-induced macular toxicity

**DOI:** 10.3205/oc000135

**Published:** 2020-02-27

**Authors:** Parveen Sen, Sridharan Sudharshan, Aniruddha Banerjee, Abhinav Dhami

**Affiliations:** 1 Department of Retina, Shri Bhagwan Mahavir Vitreoretinal Services – Sankara Nethralaya, Chennai, India; 2 Medical Research Foundation, Chennai, India; 3 Department of Uvea & Intraocular Inflammation, Sankara Nethralaya, Chennai, India; 4 Elite School of Optometry, Chennai, India

## Abstract

Efavirenz (EFV), a non-nucleoside reverse transcriptase inhibitor, is commonly used to treat HIV-infected individuals. We report a case of painless, progressive and bilateral blurring of vision in an HIV-positive 54-year-old lady within months of treatment with anti-retro viral therapy including Efavirenz. On presentation, her visual acuity was 6/18; N24 and 6/9; N10 in both eyes with mottling of the retinal pigment epithelial at the macula with corresponding scotomas on HVF 30-2 and loss of ellipsoid layer on spectral domain optical coherence topography (OCT). Though full field ERG was normal, multifocal ERG revealed reduced foveal and parafoveal amplitudes. Our case emphasizes the need of periodic ocular examination of these patients on long-term EFV.

## Introduction

Use of Efavirenz (EFV), the first generation non-nucleoside reverse transcriptase inhibitor (NNRTI) as a component of first-line antiretroviral therapy (ART), has been accepted worldwide [1]. Though EFV increases CD4 counts, it is known for a high rate of neuropsychiatric adverse events like dizziness, insomnia, impaired concentration, and hallucinations [2]. Side effects of Efavirenz usually begin within the first few days of therapy and resolve after 2–4 weeks, but 10% of patients have complaints even after discontinuing the drug [3]. Ocular toxicity has rarely been reported. We report clinical and electrophysiological characteristics of a case of EFV-related macular toxicity in an HIV patient who had been on the drug for 9 months.

## Case description

**Figure 1:**
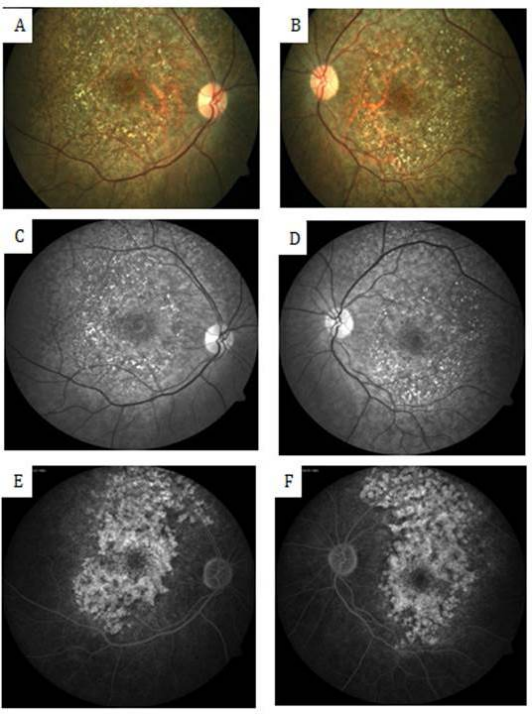
A, B) Retinal pigment epithelium mottling around the macular and para-macular area in fundus photo C, D) Dark patches at the macular region in fundus autofluorescence (FAF) E, F) Hyperfluorescence observed at macula and surrounding macular region in fundus fluorescein angiography (FFA)

**Figure 2:**
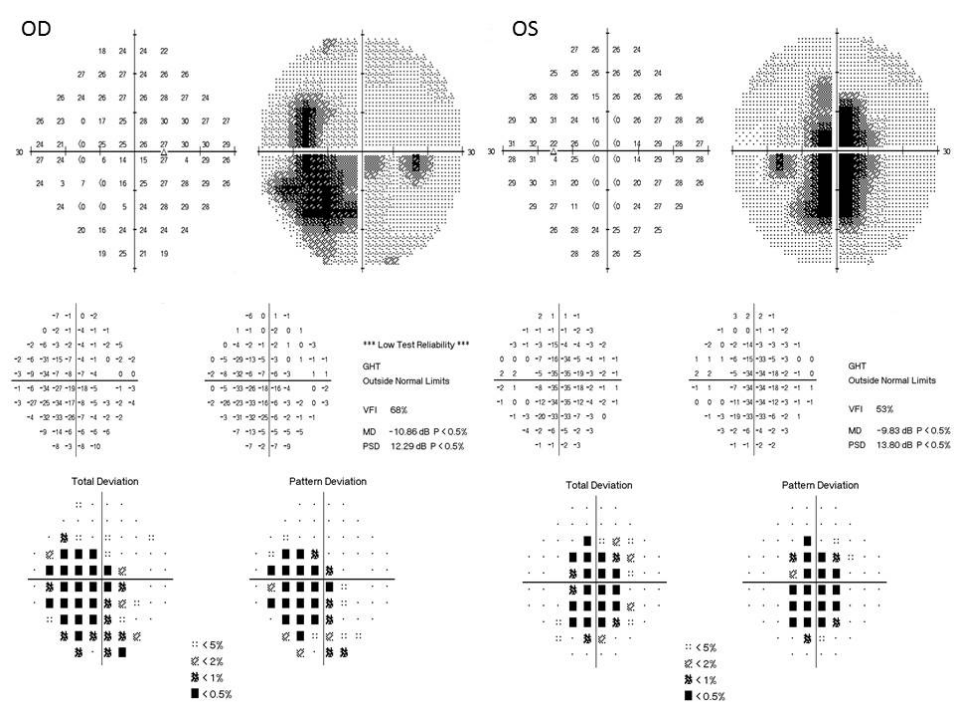
Central and paracentral field defects in both eyes in 30-2 Humphery visual fields (HVF)

**Figure 3:**
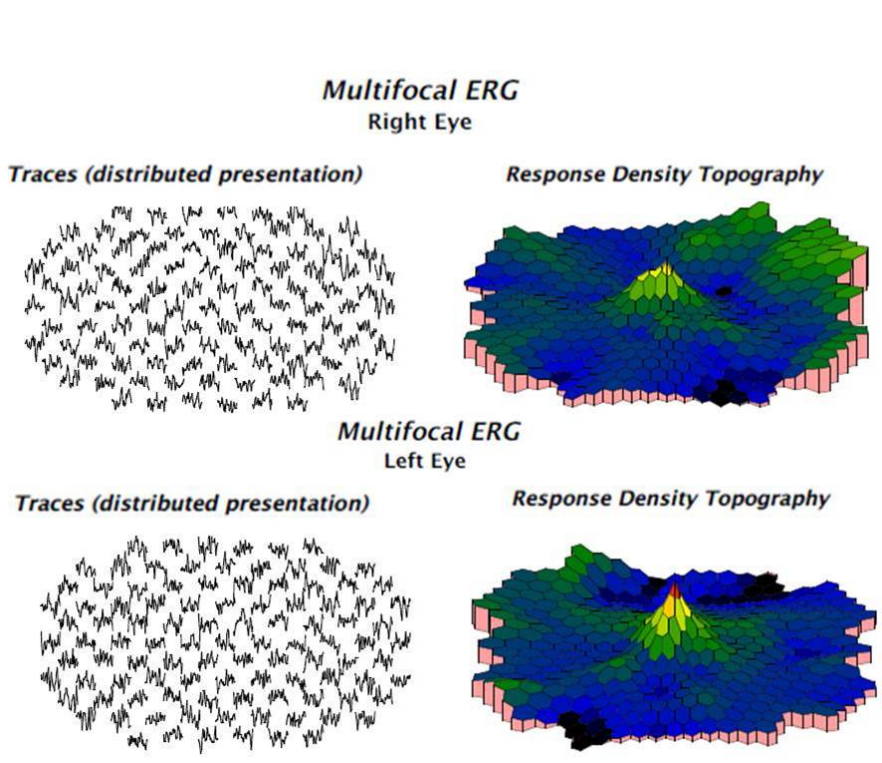
Reduced foveal, parafoveal and perifoveal responses noted in multifocal electroretinogram (MFERG)

**Figure 4:**
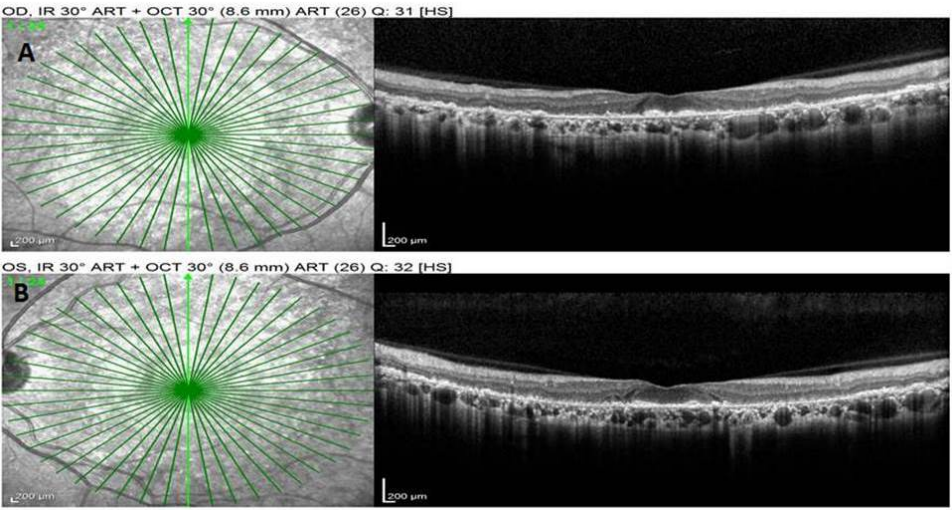
Spectral domain optical coherence tomography (SD-OCT) showed complete loss of the ellipsoid layer and external limiting membrane, although inner retinal layers were intact.

A 54-year-old female known to haven been HIV-positive for 8 years presented to us with the chief complaints of painless progressive diminution of vision in both eyes, more so in the right eye. She was a hypothyroid and hypertensive under medication with good control. She had been under treatment for HIV infection for 8 years and was currently on Efavirenz 600 mg, Lamivudine 300 mg and Tenofovir disoproxil fumarate 300 mg. Efavirenz had been started by the treating physician 9 months ago. The patient started complaining of symptoms of blurring of vision a few months after starting EFV. There was no history of trauma or nyctalopia. She did not give history of a similar problem in the family or any previous ocular problems. At presentation, her best-corrected visual acuity (BCVA) was 6/18, N24 in right eye and 6/9, N10 in the left eye. Anterior segment evaluation as well as intraocular pressure was normal. No relative afferent pupillary defect was seen in either eye. Fundus evaluation showed discrete areas of retinal pigment epithelial (RPE) mottling at the posterior pole in both eyes in an annular manner just sparing the fovea. The optic disc and retinal vessels were normal. Fundus photo (FF 450Plus with Visupac, Zeiss, USA) showed RPE mottling around the macular and para-macular area (Figure 1 A, B (Fig. 1)). Fundus autofluorescence (FAF) (FF 450Plus with Visupac, Zeiss, USA) revealed discrete dark patches at the macula in both eyes corresponding to the area of RPE mottling suggestive of RPE atrophy (Figure 1 C, D (Fig. 1)). Fundus fluorescein angiography (FFA) (FF 450Plus with Visupac, Zeiss, USA) showed hyperfluorescence at the macula and the surrounding macular region in both eyes (Figure 1 E, F (Fig. 1)). Peripheral retina was normal. The 30-2 Humphery visual fields (HVF) (HFAII 750, Carl Zeiss, Germany) showed central and paracentral field defects in both eyes (Figure 2 (Fig. 2)). Multifocal electroretinogram (MfERG) (Veris FMS II, Electro-Diagnostic Imaging, CA, USA) showed reduced N1 and P1 amplitudes in foveal, parafoveal, and perifoveal ring responses (Figure 3 (Fig. 3)). The maximum reduction of amplitudes was seen in the parafoveal responses even as the foveal peak was maintained. Spectral domain optical coherence tomography (SD-OCT) (Spectralis OCT, Heidelberg Engineering, Franklin, USA) revealed an almost complete loss of the ellipsoid layer and external limiting membrane especially in the parafoveal area with a small island of preserved photoreceptors at the fovea. The inner retinal layers were unaffected (Figure 4 (Fig. 4)). 

## Discussion

ART is known to cause a wide range of toxicities and drug-related adverse reactions [4]. HIV infection itself is associated with many ocular manifestations including microangiopathy and cytomegalovirus retinitis. Retinal toxicity due to other ART drugs such as Didanosine (DDL), Clofazimine and Ritonavir have been reported to cause damage to the retinal pigment epithelium (RPE) [5], [6]. DDL toxicity affects both children and adults with changes in ERG and HVF [6]. More than 90% of EFV is metabolized in the liver by cytochrome P450 (CYP) 2B6 into the major metabolite 8-Hydroxy Efavirenz which has been shown to be 10-fold more toxic than EFV to the neuronal cells at the concentration seen in cerebrospinal fluid after therapeutic doses [7]. There have been reports of no ocular toxicity with EFV even after prolonged use [8]. Wide inter-patient variability in the toxicity due to EFV can be explained by the variability in pharmacokinetics due to polymorphisms in cytochrome P450 CYP2B6, which is the major metabolizing enzyme [9]. Increased risk of toxicity due to EFV has been seen in females and non-Caucasians; both these risk factors were there in our patient as well [10]. Literature reporting retinal toxicity with Efavirenz is very limited, with none in the Indian population. In view of limited literature supporting the retinal toxicity due to Efavirenz [6], this case report provides insight on the possible correlation of EFV with macular toxicity with multimodal imaging localizing the site of damage to the RPE especially in the parafoveal region within months of starting the medication. Annular distribution of EFV retinal toxicity has also been reported by Pereira et al. [5].

## Conclusion

Close follow-up of patients on Efavirenz is necessary so that permanent visual loss can be avoided by timely cessation of the drug.

## Notes

### Competing interests

The authors declare that they have no competing interests.
